# Policy levers and priority-setting in universal health coverage: a qualitative analysis of healthcare financing agenda setting in Kenya

**DOI:** 10.1186/s12913-020-5041-x

**Published:** 2020-03-06

**Authors:** Tessa Oraro-Lawrence, Kaspar Wyss

**Affiliations:** 1 Swiss Center for International Health, Swiss Tropical and Public Health Institute, P.O. Box 4002, Basel, Switzerland; 2 University of Basel, Basel, Switzerland

**Keywords:** Kenya, UHC, Priority-setting, Health financing, Health policy

## Abstract

****Background**:**

Competing priorities in health systems necessitate difficult choices on which health actions and investments to fund: decisions that are complex, value-based, and highly political. In light of the centrality of universal health coverage (UHC) in driving current health policy, we sought to examine the value interests that influence agenda setting in the country’s health financing space. Given the plurality of Kenya’s health policy levers, we aimed to examine how the perspectives of stakeholders involved in policy decision-making and implementation shape discussions on health financing within the UHC framework.

****Methods**:**

A series of in-depth key informant interviews were conducted at national and county level (*n* = 13) between April and May 2018. Final thematic analysis using the Framework Method was conducted to identify similarities and differences amongst stakeholders on the challenges hindering Kenya’s achievement of UHC in terms of its the optimisation of health service coverage; expansion of the population that benefits from essential healthcare services; and the minimisation of out-of-pocket costs associated with health-seeking behaviour.

****Results**:**

Our findings indicate that the perceived lack of strategic leadership from Kenya’s national government has led to a lack of agreement on stakeholders’ interpretation of what is to be understood by UHC, its contextual values and priorities. We observe material differences between and within policy networks on the country’s priorities for population coverage, healthcare service provision, and cost-sharing under the UHC dispensation. In spite of this, we note that progressive universalism is considered as the preferred approach towards UHC in Kenya, with most interviewees prioritising an equity-based approach that prioritises better access to healthcare services and financial risk protection. However, the conflicting priorities of key stakeholders risk derailing progress towards the expansion of access to health services and financial risk protection.

****Conclusions**:**

This study adds to existing knowledge of UHC in Kenya by contextualising the competing and evolving priorities that should be taken into consideration as the country strategises over its UHC process. We suggest that clear policy action is required from national government and county governments in order to develop a logical and consistent approach towards UHC in Kenya.

## Background

Priority-setting is a central part of building efficient, responsive and resilient healthcare systems [1, 2]. In many low- and middle-income countries (LMICs), efforts to strengthen healthcare planning and delivery systems are often complicated by a plethora of epidemiological, social, economic, and administrative challenges. These issues necessitate difficult choices on which health actions and investments to fund: decisions that are complex, value-based, and highly political. Priority-setting aims to provide the best use of financial and other resources in line with population value choices, demand, and need. This government-led process theoretically allows a diverse range of healthcare stakeholders to articulate their preferred values and agendas in order to achieve consensus on the direction for a country’s health agenda [3]. In practice, however, healthcare decision-making in many LMICs is often ad-hoc resulting in inefficient and inequitable resource allocation [4].

Against this backdrop, universal health coverage (UHC) has been identified globally as a unifying platform for countries’ health systems development [5]. It is defined as the aspiration of a country’s citizens to obtain access to essential health services based on need without the risk of financial hardship. UHC is ultimately a progressive and aspirational goal which is characterised by the achievement of equity through three key dimensions: population coverage; service coverage; and cost-sharing [6]. By focusing on these crosscutting objectives, it aims to provide a holistic strategy for tackling the formidable health systems challenges faced across a variety of settings, as highlighted within the global Sustainable Development Goals (SDGs) [7].

While most stakeholders agree on the basic definition of UHC, a diversity of practical interpretation has emerged reflecting the differing perspectives of each country’s unique social, political, economic and epidemiological realities [8]. This plurality of interpretation makes it necessary for governments to steward a participative decision-making process through which a defined approach towards UHC may be developed [1, 3, 9]. In spite of this, many governments have maintained a haphazard approach towards health systems priority-setting, resulting in arbitrary and inconsistent planning decisions [10, 11]. In Kenya, where UHC is mentioned conceptually in the national government’s health policy documents and medium-term development agenda, there remains limited articulation of the explicit choices and trade-offs to be considered in steering the country’s UHC policy direction [12–14]. While efforts were previously made to define the country’s UHC path through the country’s Health Financing Strategy 2016–2030, this document has remained unpublished with seemingly limited impact on the health system’s strategic direction [15]. There is therefore an urgent need to identify the key considerations underpinning Kenya’s UHC agenda, as well as the divergences that may limit the success of these endeavours. This paper seeks to investigate the fundamental priorities driving provisions towards UHC in Kenya. In doing so, we examine the perspectives of key health systems stakeholders on the policy considerations for health financing strategy in Kenya.

In order to understand Kenya’s UHC ambitions, it is first necessary to identify the specific systems-wide approaches undertaken under the UHC banner. The national government has sought to promote interventions that apply six key principles highlighted within the current Kenya Health Policy: equity; people-centredness; participation; multi-sectoralism; efficiency; and social accountability [12].

In terms of health service coverage, the Kenyan Government has promoted investments that indirectly encourage equity, people-centredness and participatory approaches. Accordingly, healthcare management in the country was decentralised to county level in 2012 in order to reduce regional disparities in health outcomes and increase responsiveness to the unique epidemiological and social contexts inherent within each of the country’s 52 counties [16–18]. This transfer of responsibility for healthcare planning and budgetary allocation from national to sub-national level has effectively tasked county governments with ensuring the successful implementation of Kenya’s health policy. This has created a need for financial and technical empowerment and capacity building at sub-national level in order to effectively carry out its new role.

In addition, the national government expanded service offerings within the country’s national health insurance scheme, the National Hospital Insurance Fund (NHIF), in order to improve access to healthcare services. The NHIF is the largest health insurance provider in Kenya, covering 88.4% of the insured population [19]. Established in 1966, it operates as a contributory scheme, with mandatory coverage of the formal sector through direct taxation of salaries and voluntary enrolment of the informal sector. As a result, the Scheme has achieved a near-universal coverage of the country’s formal sector, while informal sector coverage has remained low at 18.9% [20]. The NHIF provides inpatient and outpatient services to all enrolees, with a defined comprehensive package for the formal sector, and an evolving benefit package for the informal sector.

In order to improve the country’s health service and population coverage, the NHIF has launched three national government-funded flagship programs since 2012: the *Linda Mama* programme which aims to provide free maternity services to all Kenyan women; the Health Insurance Subsidy for the Poor (HISP) which seeks to provide free comprehensive health insurance coverage to 9 million indigents by 2020; and the *Inua Jamii* programme which aims to provide free comprehensive health insurance coverage to the elderly and those with physical disabilities. The government has also sought to encourage social accountability by hiring local agents such as community-health workers (CHWs) to conduct NHIF population sensitisation and targeting activities [21]. Through this process, it aims to convince informal sector members to join the NHIF, as well as to identify indigents who may benefit from the full subsidisation of their NHIF premiums.

In spite of these policy ambitions, Kenya’s government has been unable to implement a long-term financial strategy to support its UHC direction. Indeed, we note a lack of definition of the values and trade-offs competing for health resources within Kenya’s limited fiscal space. This makes it difficult to implement meaningful resource and strategic investment into health policy goals, and limits the potential impact of actions and investments towards UHC in Kenya [13, 22, 23].

Given the intrinsically participative nature of health priority-setting, the inclusion of a broad range of stakeholders is essential for the success of UHC in the Kenyan context [24]. Indeed, the multiplicity of stakeholder interests and values has been highlighted in various studies investigating the political economy of various health systems reforms in the Kenyan healthcare sector [16, 25, 26]. As such, it is imperative to identify and consider the ideological positions of key policy stakeholders when considering Kenya’s ideal path towards UHC. This study seeks to examine the perspectives of key stakeholders on the policy considerations that influence agenda setting in the country’s health financing space. Given the plurality of Kenya’s health policy levers, we aim to assess how stakeholders involved in policy decision-making and implementation perceive health financing considerations within the country’s universal health coverage (UHC) framework. We further seek to understand how viewpoints within and across policy groups may influence how health financing priorities are set within the country.

## Methodology

### Study setting

This manuscript reports the analysis of a series of in-depth key informant interviews at national and county level (*n* = 13) carried out between April and May 2018. The interviews targeted national- and county-level policymakers with specialist knowledge on Kenya’s health priority-setting process in order to identify the fundamental values related to provisions for population coverage of UHC in Kenya; the range, scope and quality of health care service provision; and the investments necessary for reducing the financial impact of ill health amongst the Kenyan population.

Most health actors agree that the UHC process is ultimately a trade-off between investments in three critical areas of a health system: population coverage; service coverage; and financial protection ( [6]. Given that the above-mentioned UHC dimensions have received near-universal backing amongst World Health Organization member states [5], we applied them to the study design in order to rationalise discussions on the country’s health systems priorities. This approach, in our view, was more likely to present a holistic perspective of the values and trade-offs to be considered in the push towards UHC.

National stakeholders were stratified into three policy circles or networks: national government stakeholders responsible for defining national health financing policy in the country; development partner stakeholders who provide both financial and technical support on health systems strategies to the national and county governments, and the technical experts advising both groups. County-level stakeholders included current and former Chief Health Officers responsible for formulating and implementing county health strategies in Kisumu County.

The presented research was undertaken in Nairobi – where most national stakeholders and technical experts are domiciled – and Kisumu. Kisumu is the third largest city in Kenya with a population of 491,893 individuals [27]. The key informant interviews at county level were embedded within a larger quantitative study in Kisumu, which aimed to investigate the factors influencing voluntary enrolment into the NHIF amongst residents of urban informal settlements [28].

### Data collection

Thirteen in-depth interviews were conducted using a semi-structured discussion guide. Each interview lasted approximately 45 min, and elicited discussions on stakeholder value interests in Kenya’s health financing space. In order not to pre-empt the value interests of respondents, the direction of conversation was steered as much as possible by preceding interviewees’ responses. The interview guide is provided as an appendix to this manuscript.

Of the thirteen interviews conducted, three were national-level government stakeholders, two were county-level government stakeholders, three were development partners, and five were technical experts. Of the technical experts, two had played a role providing technical expertise to the NHIF directly, two had advised the Ministry of Health on its universal health care strategy directly, and three had played a role advising development partners at the time of the study. One of the development partners had previously been a national government policymaker in the five years preceding this study. See Table 1.

**Table 1:** Overview of key informants and where they worked (health system level)

Research Phase	Total
In-depth interviews with national-level government stakeholders	3
In-depth interviews with county-level government stakeholders	2
In-depth interviews with development partners	3
In-depth interviews with technical experts	5
**Total**	**13**

### Data analysis

Ten of the thirteen interviews were audio recorded and summary notes were taken. Audio files were transcribed verbatim. Three interviews were not recorded with respect to interviewee requests, but detailed notes of the interviews were taken. Data were subsequently analysed using the Framework Method, which enables comparison of emerging themes across different policy groups through inductive and deductive approaches [29]. This method was applied in this study using the following approach: (i) the transcribed data were compared with the audio files in order to ensure their veracity; (ii) the audio recordings were played severally in order to ensure familiarisation with their contents and themes; (iii) open coding of the transcripts was conducted in MaxQDA in order to categorise a preliminary set of themes based on the key UHC building blocks of population coverage; service coverage; and financial protection; (iv) iterations were made to the coding system as new themes emerged from the interview data; and (v) emerging themes were analysed across individual interviewees and policy groups using MaxQDA analysis functions.

The final thematic analysis focused on interpreting similarities and differences amongst stakeholders on the challenges facing Kenya’s health system that hinder the achievement of UHC in terms of its three building blocks; potential solutions to the problems identified; and the political and real-world situation that aided or hindered the achievement of the articulated solutions.

## Results

### Universal health coverage in Kenyan context

Key informants representing the different stakeholder groups viewed UHC as a complex process within which a variety of players, structures and concerns should be incorporated. While there was congruence on the multi-faceted nature of UHC, several stakeholders accused the national government of lacking a holistic approach towards UHC, suggesting that it often limited its interventions to healthcare financing through the NHIF:

*“… the main agenda has not always been about providing comprehensive public information about what UHC is about. If you go to the Ministry of Health [they] just tell you to enrol with NHIF.” (Development partner).*

### Population coverage

#### Definition of target UHC population

Kenya’s national health strategy to date has largely focused on the achievement of core health targets amongst specific populations such as mothers, children, and the elderly [30]. In line with this, our interviews revealed a lack of consensus on the definition of the population to be covered with essential health services in order to achieve UHC. When queried, governmental and non-governmental respondents oscillated between defining population coverage as providing UHC to the whole Kenyan population; at least 80% of the population; and focusing solely on the poorest Kenyans. In spite of this variance, we noted convergence amongst stakeholders on the role of the NHIF as the organisation responsible for pooling population groups in order to facilitate equitable access to healthcare services in Kenya.

Stakeholders identified two target population groups as integral to efforts on optimising the NHIF under the UHC banner: the informal sector and indigents. Governmental and non-governmental respondents acknowledged concerns about the low enrolment numbers amongst these population groups, citing difficulties in market segmentation. This, they intimated, was due to the mutability of these populations and weak household identification systems within the country. In order to mitigate these concerns, stakeholders submitted that the actions of the national government, county governments, and individual informal sector members were key to the success of the NHIF. The degree to which each group was held responsible for increasing population coverage within the NHIF, however, varied across policy networks.

#### Role of national and county government in population coverage

Reflecting the transfer of responsibility for healthcare provision and management to the sub-national government in Kenya’s Constitution [31], most interviewees expressed strong expectations that the county government would play a central role in identifying and targeting households for enrolment into the NHIF. While acknowledging their role in identifying indigents, county stakeholders felt that the national government should provide the financial resources for increasing population coverage through the NHIF.

*“The national government should first tell us how much money they have for UHC… if they are going to cover the indigents, then the county does not need to worry about that.” (County stakeholder).*

Respondents across policy networks acknowledged the NHIF’s efforts to improve the process of population targeting by engaging community health workers (CHWs) – who make up the first level of the Kenyan healthcare system – to sensitise and register all local households in a number of pre-selected counties. Given the positioning of CHWs under the county government system, respondents emphasised the need for counties to develop strong, sustainable CHW payment policies. It was noted that counties varied in their CHW payment policies: some paid CHWs directly, while others relied on donors for their payment. This, in stakeholders’ view, raised the possibility of donor priorities superseding government UHC strategy, thereby weakening the authority of governments to effectively use CHWs for implementing official Kenyan health policy.

*“… there are some counties which are paying stipends [for NHIF advocacy work] already, others are not yet there. So the Community Health Volunteers are left to rely on other partners who come in with activities that are engaging them and then they get the lunch, transport reimbursement.” (Development partner).*

Few concrete solutions were offered on how to better facilitate the role of county governments in targeting and enrolling residents into the NHIF, highlighting the complexity of population coverage in Kenya. However, some general suggestions offered by stakeholders ranged from the use of innovation for population mapping to the complete integration of the NHIF into government services to enforce NHIF premium payment.

*“There should be some legal provisions to put some level of discipline on the population. For example, you can tie access to government services to NHIF enrolment…” (Technical expert).*

#### Individual informal sector households

In light of the difficulties in enforcing mandatory NHIF coverage in Kenya due to ineffective household identification strategies, stakeholders across policy groups conceded that individual households would continue to play a major role in NHIF’s risk-pooling strategy.

Stakeholders showed unanimity in their reservations about the NHIF’s ability to effectively attract voluntary members. Some stakeholders considered financial instability as a key barrier to voluntary NHIF enrolment and expressed concerns about the equity implications of targeting heterogenous informal sector groups.

*“Let’s say we have two informal sector members who [provide motorcycle transport]. How will you be able to identify who [is in a financial position to afford NHIF]?” (County stakeholder).*

Limited solutions were offered for the underlying inequity within the NHIF’s population coverage, with respondents diverging on who ought to receive NHIF premium subsidisation. We did not identify any clear patterns on this issue within or across policy networks, with many stakeholders offering no opinion on the topic.

*“The question is, do we request the government to finance all households within the informal sector, or do we provide incentives or partial subsidies so that people can join the NHIF?” (Development partner).*

Another point of discussion was what stakeholders viewed as the complexity of financial decision-making amongst those with limited disposable income: in their view, the decision to enrol into the NHIF was complicated by a low NHIF value perception amongst informal sector members and ineffective NHIF outreach mechanisms. Technical stakeholders expounded on the need to simplify communication targeting informal sector members, urging the targeting of specific population groups such as women, churches, and savings groups as a means of expanding voluntary NHIF enrolment.

“*For UHC to be achieved, we need to cognizance of the fact that communities don’t have the same knowledge as we do, public information activities have to happen at all levels.*” *(Technical expert).*

However, we were unable to identify through our interviews under whose purview the creation and dissemination of these sensitisation materials would fall.

A small proportion of development partners and technical experts deplored what they viewed as obsolete enrolment procedures, such as requiring birth and marriage certificates for enrolment. They noted that this disregards the evolution of Kenyan societal norms by underestimating the difficulties faced by underprivileged communities in getting official documentation.

“*The NHIF has policies that are so archaic, such as availing marriage certificates. Go to slum areas… you’ll find women of reproductive age who have multiple children but no identification card, much less a marriage certificate*.” (Technical expert).

It was further noted that perceived punitive measures for defaulting on premium payment disincentivised NHIF uptake. These concerns were, however, in the minority.

### Service coverage

A majority of respondents felt that the priority of UHC in the context of service coverage was to provide adequate and quality healthcare services to the general public. There was, however, divergence on how this would be achieved in Kenya. Indeed, the country’s health strategy largely focuses on systems strengthening through the achievement of specific core national health targets, such as HIV, maternal and child health indicators [30, 32]. There is currently no defined benefit package provided by the national government beyond these health indicators. In the context of our study, respondents mentioned two areas of focus as the main policy levers that directly impacted service coverage in Kenya: healthcare service planning and provision.

#### Healthcare service planning

Stakeholders generally felt that health system needs were highly county-specific, noting the different levels of investment across the country.

*“… Each county has very specific needs. Some struggle with equipping facilities. Others have problems with the distribution of facilities, while others have issues with how facilities are managed.” (Technical expert).*

In spite of this, the respondents generally agreed that healthcare service provision across the country was inadequate, highlighting concerns about limited investment in infrastructure and the human resource base. Some NHIF and technical expert respondents deplored what they viewed as misplaced healthcare investment decisions at national level.

*“Instead of buying high-end equipment, instead of doing all these fancy, politically visible, expensive things… they should be building more dispensaries, more health centres, making sure that everyone is within five kilometres of a health centre” (Technical expert).*

Further, some respondents expressed frustration with county governments’ unwillingness to allocate funds collected at health facilities towards improving local health systems. Several county government stakeholders and technical experts felt that many county governments often paid lip service to UHC, focusing on enrolling residents into the NHIF but not on providing the required capital investment into healthcare facilities. This strategy, in their view, was counterproductive and risked exacerbating poor healthcare service provision at the local level.

Concurrently, while acknowledging efforts to increase the population’s access to healthcare services through the NHIF, stakeholders across the policy networks expressed concern that there had been insufficient focus on increasing the number of NHIF-empanelled healthcare facilities that target under-served populations.

*“Those in pastoralist and sparsely-populated communities may have to travel up to seventy kilometres to access healthcare services even after paying for NHIF. That doesn’t add up” (Development partner).*

Currently, the NHIF conducts strategic purchasing by empanelling healthcare facilities to provide a defined benefit package. Health facilities are contracted under three categories by the NHIF based on their ability to provide a defined list of services [33]. Respondents acknowledged that the requirements for NHIF empanelment were often too high to be achieved by lower-level healthcare facilities, which were over-represented in rural areas. This in turn led to an imbalanced system where healthcare facilities in urban and wealthier areas were over-represented on the NHIF-approved facilities list.

Further, while some counties were collaborating with the NHIF to increase empanelled facilities, several respondents deplored what they viewed as political interference in the empanelment process. It was intimated that some counties had lobbied for lower-quality facilities to be empanelled by the NHIF, which had led to concerns about maintaining the quality of care in NHIF-approved facilities. In order to mitigate this, respondents suggested strengthening the NHIF accreditation and quality improvement system*.* Details on how this would be achieved were, however, scarce.

In order to mitigate concerns on the resilience and adaptability of local healthcare service provision, several technical experts proposed that the national and county governments work to create distinct public-private healthcare networks with defined roles. County stakeholders also highlighted the integral role of preventative health in relieving pressure on the healthcare delivery system, suggesting that:

*“We must put a lot of emphasis on prevention of these diseases… A lot of diseases that we have in this region are communicable diseases.” (County stakeholder).*

#### Healthcare service provision

When probed about Kenya’s essential health benefit package, we observed a lack of consensus in the views expressed by the different policy groups. While a majority of development partners and technical experts lamented what they viewed as a broad and arbitrary health benefit package, most NHIF and county stakeholders expressed satisfaction with the current voluntary NHIF health benefit package. All stakeholders tasked the Ministry of Health with the responsibility of applying strong technical expertise in order to define and cost a realistic minimum health benefit package that could be offered on a large scale in the country.

*“The Ministry of Health must own the process of defining the [minimum] health benefit package, regardless of who is financing it. We must not allow [other parties] to dictate to them” (Technical expert).*

We observed that several stakeholders did not distinguish between the Kenya Essential Package for Health Services (KEPHS) – which highlights the universal minimum entitlements to be provided to all Kenyans in an equitable manner – and the NHIF health benefit package during the course of the interviews. Only a single technical expert explicitly articulated the need to definitively consider which KEPHS entitlements could realistically be offered through the NHIF, given the exclusion of several levels of healthcare providers from the NHIF-selected facilities.

Stakeholders who expressed concerns about the sustainability of Kenya’s health benefit package attributed their unease to political interference by the country’s President, and technical incapacity at the Ministry of Health.

*“The issue at the Ministry of Health has to do with knowledge and capacity… and is probably what has led to fragmentation of approaches on the implementation of UHC” (Development partner).*

Technical expert and development partner respondents articulated the need to revise the country’s minimum health benefit package in line with the financial realities and epidemiological profile of the country, as well as a strong evidence base of cost-effectiveness, risk, and equity. They further suggested that the private sector’s role lay in providing top-up insurance over and above the minimum health benefit package.

### Cost-sharing and user fees

Current Kenyan national health policy highlights the reduction of out-of-pocket payment as a key health indicator [12, 15, 30]. However, the level of cost-sharing remains undefined within official documents. In light of this, although most respondents expected UHC to reduce out-of-pocket payments for the Kenyan public, our findings uncovered a disconnect between stakeholders on the level of cost-sharing acceptable for receiving healthcare services in Kenya. While all county stakeholders interviewed envisaged co-payments by members of the public ceasing in their entirety under the UHC framework, some technical experts suggested that maintaining a moderate level of co-payment would help in controlling moral hazard in Kenyan hospitals. It was not clear which services respondents expected would be subject to co-payment, although one respondent suggested limiting them to services beyond the minimum health benefit package.

According to stakeholders, cost-sharing in the Kenyan context is largely executed at facility level, with responsibility falling upon three major players: the national government; county governments, and the NHIF. A minority of technical experts advocated for a tax-based health financing system, citing the implausibility of achieving UHC through the NHIF. Several technical experts opined that the national government’s role in cost-sharing had been negated by the 2012 Constitution, which devolved most healthcare functions to the sub-national level. Nevertheless, they noted that the national government still had a major role to play in revenue raising for the sub-national level and subsidising the NHIF.

*“Counties don’t have a leeway to source for financial support from outside countries without going through the national government. So the national government remains a key instrument.” (Development partner).*

Indeed, it was observed that the availability of healthcare funding at county level was limited: according to the Kenyan Public Finance Management Act, funding collected from local healthcare facilities is required to be pooled in a general county revenue fund account with no obligation for its subsequent allocation to health services [34]. Given the pervasive link between county budgets and medium-term political goals, respondents expressed concern about the sustainability of revenue raising for health at sub-national level.

*“You need to institute the financing for longevity. So you even need to enact policies that can make sure that this money whether the current governor is there, this money continues to be availed. It [cannot be reliant on] partners, because partners have a shelf life.” (Technical expert).*

When queried about their concerns on cost-sharing in Kenya’s health system, stakeholders across policy networks observed that the stability of resource mobilisation for health would determine the level and sustainability of cost-sharing measures undertaken. At the national level, there was consensus that the diminishing healthcare funding was a major risk to revenue stability, linking it both to reduced donor funding as well as reduced government funding to the NHIF. NHIF stakeholders noted that national budget allocations to health had not matched political rhetoric on expanding the organisation’s population and service coverage. In order to navigate the reduced national financial allocations to health, several NHIF stakeholders and technical experts endorsed the pooling of parallel funding sources into the NHIF in order to increase its resource base. Some county and technical expert stakeholders also suggested aligning the minimum universal health entitlements to the financial constraints of the national government, with one technical expert stating:

*“We need to figure out our ability to mobilise resources… Following that, we need to determine the range of services that … we can offer for free.” (Technical expert).*

County stakeholders expressed frustration about what they viewed as the offloading of healthcare functions to county level without the necessary financial support being provided by national government. As an example, respondents pointed towards the national government’s purchase of unnecessary medical equipment, which they felt overstepped its governance role and diverted much-needed funds from the health system. Concurrently, NHIF stakeholders and technical experts felt that county governments had not used allocated health funds efficiently, pointing towards the return of allocated health funds to the National Treasury at the end of the financial year. Respondents blamed a *“lack of understanding of budgeting processes”* and *“lack of accountability and efficiency”* for these problems*.*

At facility level, stakeholders across policy networks considered the availability of NHIF funding as integral in aiding cost-sharing processes. A number of technical experts noted the lack of agreement on financing modalities to facilitate co-payment at facility level. Respondents further noted NHIF reimbursement processes that they viewed as unfair to public facilities, with a county stakeholder opining:

*“… NHIF reimbursements do not come as quickly as those for private facilities… My assumption is those who can [pay a bribe] get their reimbursement faster” (County stakeholder).*

These responses amplified concerns punctuated throughout our interviews about corruption within the NHIF. NHIF stakeholders did not however, share these concerns.

## Discussion

In this study, we sought to investigate the values and priorities underpinning Kenya’s path towards UHC. In doing so, we examined the perspectives of key health systems stakeholders on the policy considerations for UHC and its potential impact on the country’s health financing decisions.

It is clear from the outset that Kenyan stakeholders recognise UHC as a major goal in the country’s health policy and priority-setting landscape. While the robust dialogue within Kenya’s health policy circles signals intentionality to create a path towards UHC, our findings suggest that the country lacks a centralised, systematic and inclusive process through which this agenda can be driven. As a result, a highly dynamic policy environment has emerged where actors differ substantively in their interpretations of the country’s UHC values and priorities. This reflects existing priority-setting studies in Kenya that have found variances in the way different stakeholders perceive health systems decisions in the country [16, 26]. We postulate that stakeholders’ articulated values are aligned to their dogmatic principles and their depth of interaction with the country’s health and political systems. Accordingly, our findings suggest that county and national government stakeholders prioritise short-term health maximisation as their main UHC policy goal in Kenya, ostensibly due to their proximity to elected government officials. Conversely, technical experts seem to value the legitimacy of their policy decisions, pushing for objectives that, in their view, optimise technical feasibility and sustainability. Development partners, on the other hand, leverage their fiduciary obligation to funders when participating in priority-setting activities.

Conflicting policy positions aside, progressive universalism has emerged as the preferred approach towards UHC in Kenya, with most interviewees prioritising an equity-based approach towards healthcare service access and financial risk protection. This strategy is particularly pertinent given the regressive nature of household healthcare contributions in Kenya, as well as the impact of developmental, financial and epidemiological inequity on the health system’s responsiveness and resilience [35–37]. It further reflects the global consensus towards a pro-poor UHC approach that prioritises equity and equality, and underlines a commitment towards a more holistic healthcare approach under the UHC banner [1, 38–41]. We posit that stakeholders in Kenya are particularly supportive of systemic health financing and service delivery measures that counterbalance geographic and socioeconomic inequities in the Kenyan healthcare system.

While support for progressive universalism seems unequivocal amongst key health systems stakeholders in Kenya, it is likely that their conflicting priorities will continue to complicate progress towards UHC. We hypothesise that the lack of strategic leadership from Kenya’s national and county governments risks derailing progress towards the expansion of access to health services and financial risk protection. Indeed, the WHO suggests that strong stewardship from governments is key to achieving UHC in each individual country [1, 6, 9, 42].

In terms of population coverage, our findings suggest that most stakeholders recognise the NHIF as central to Kenya’s efforts in expanding population risk pooling. We, however, observed divergence amongst stakeholders on two priority issues: systemic support for a robust population identification mechanism; and clarity on stakeholder roles in the financial coverage of priority population groups. Non-governmental stakeholders, in particular, were apprehensive about NHIF efforts to effectively identify informal sector members for enrolment, highlighting inefficiencies in its current population identification and means-testing mechanisms. Compounding these perceived inefficiencies is the unwillingness of county and national government stakeholders to commit towards providing long-term financial support for the protection of indigents from the undue financial pressure of ill health. This goes against WHO recommendations that place the burden of providing access to underserved communities on governments, and threatens the success of Kenya’s risk pooling efforts [11]. Our findings further reiterate the need for substantive support towards the subsidisation of health costs for vulnerable groups.

When queried about health service coverage, we observed discordance between policy networks on the range of healthcare services to be provided to the Kenyan population as part of the country’s UHC efforts. Stakeholders’ priorities were highly dogmatic, with government stakeholders preferring a broad set of healthcare services that prioritised socially-acceptable outcomes, and technical experts and development partners endorsing a limited costed essential health benefit package based on a strong evidence-based process. Our findings also revealed a lack of clarity on the role of different players in providing access to the Kenyan essential benefit package. Given that most stakeholders agreed that national and county governments, the NHIF, and private health insurance providers should play a role as healthcare service purchasers, there is an urgent need to articulate how purchasing should be split between these players in an efficient and coherent manner.

In terms of cost-sharing, we posit that the limited financial investment by national and county governments in Kenya’s health goals remains a hindrance to UHC efforts. Annual national budget documents currently show that financial contributions towards the essential health benefit package have largely stagnated contrary to the national government’s policy rhetoric [13, 22, 23]. Given that the national government bears responsibility for the coverage of the costs associated with the essential health benefit package [42], this calls into question the feasibility of effectively subsidising health costs for the Kenyan population. Further exacerbating these concerns is the perception amongst interviewees that public health facilities are discriminated against with regards to NHIF reimbursement processes. Members of the public are heavily reliant upon public facilities particularly for inpatient care [19]. We suggest that, if true, any potential discrimination against public health facilities may have a negative impact on the country’s ability of to provide comprehensive care to members of the public. We, however, hasten to note that we cannot definitively confirm stakeholders’ claims of unfair reimbursement practices against public facilities, and suggest that further research is needed in order to investigate this.

Our study also revealed concerns across stakeholder groups about the long-term financial disbursements from county governments towards existing health programmes, which were perceived to be haphazard. Technical experts, in particular, expressed frustration about the efficiency of the health budget allocation and execution at county level, with several counties returning existing funding due to systemic inefficiencies. This inefficiency, coupled with the transfer of financial autonomy to county governments risks deprioritizing health in the country. Indeed, given that the achievement of UHC is reliant upon cost-sharing for healthcare services between governments and their citizens [41], this lack of substantive commitment towards health investments by Kenya’s financial bureaucracy risks maintaining a high level of financial risk amongst the Kenyan population.

In light of the emerging gaps in interpretation of UHC in the Kenyan setting, there remain significant challenges in the country’s ability to offer accessible health services and financial risk protection to all its citizens. Unless appropriate action is taken to remedy these divergences, Kenya’s health system will continue to be regressive due to its reliance on household contributions through out-of-pocket payments [43]. It is therefore imperative for the national government to implement a strong governance system focused on defining a common and realistic set of health system values, as well as creating a strong policy, legal, institutional and regulatory framework to support the progressive achievement of UHC in Kenya.

While our study provides an important starting point for the discussion on UHC value setting in Kenya, there are several considerations that limit the generalisability of our findings. We faced difficulties in interviewing several national stakeholders and did not include public and private health service providers and community representatives, thereby missing some important perspectives on the topic under research. Nevertheless, we made the effort to interview these stakeholders’ technical advisors in order to mitigate this omission. Due to resource limitations, we limited our study at county level to stakeholders from one of the fifty-two counties. This means that while our results may be relevant across the country due to the inclusion of national stakeholders, it would be difficult to generalise the responses of county stakeholders. In spite of these limitations, we note that this is the first study in Kenya seeking to understand health financing agenda setting under the UHC umbrella.

We note that since the completion of our study, the Kenyan Ministry of Health has embarked on efforts to cost an essential health benefit package [44]. While the implementation of this evidence-based process is laudable and necessary, it is important to leverage this process against an understanding of the key value considerations that drive resource allocation in health. Indeed, it is acknowledged that technical approaches towards priority-setting are often not adhered to in the real-world setting, making it important to understand the drivers of Kenya’s health priorities outside of its budgetary, epidemiological and technical considerations [4, 10, 45].

## Conclusion

This study adds to existing knowledge of UHC in Kenya by contextualising the competing and evolving priorities that should be taken into consideration as the country strategises over its UHC process. Our research centres itself within this space by highlighting the opposing values and priorities influencing government actions and investments in Kenya’s health sector. These insights are particularly pertinent, given the need to adjust current provisions of UHC in Kenya in line with the population’s needs and realities. We suggest that clear policy action is required from national government and county governments in order to develop a logical and consistent approach towards UHC in Kenya.

Given that the Kenyan Constitution explicitly lays the purview of health system governance on the national government, the responsibility for encouraging and influencing the actions of all actors within the sector ultimately falls to its Ministry of Health. As such, our policy recommendations for national government highlight the need for leadership, communication, trust, transparency and accountability at national level, in order to foster collaboration in Kenya’s health space. We submit that four key objectives should be prioritised by the national government in order to define a common and realistic set of health system values and goals in Kenya: (i) building a robust environment for intra-sectoral collaboration to allow all health stakeholders to contribute towards UHC agenda-setting; (ii) developing and updating a viable UHC strategic vision that will drive priority actions and interventions within Kenya’s health space; (iii) developing systematic priority-setting rules through which potential UHC interventions may be objectively considered as the health system evolves; and, (iv) spearheading clear communication channels for intra- and intersectoral collaboration to optimise UHC policy adaptation and implementation, as well as resource mobilisation for health.

Additionally, we recommend four priority areas for action to empower county departments of health in their evolving healthcare service provision role: (i) identifying unique sub-national health systems priorities; (ii) developing representative county operational and implementation health plans; (iii) capacity building to improve efficiency and effectiveness in the use of financial resources; and, (iv) aligning county health plans with national health policy. These activities will be central towards assisting county governments in developing effective health budget allocation and annual planning mechanisms in order to optimise healthcare service delivery.

## Data Availability

The datasets used and/or analysed during the current study available from the corresponding author on reasonable request.

## Abbreviations

CHWs: Community Health Workers
HIV: Human Immunodeficiency Virus
KEPHS: Kenya Essential Package for Health Services
LMICs: Low- and middle-income countries
NHIF: National Hospital Insurance Fund
UHC: Universal Health Coverage
